# Looking for blood

**DOI:** 10.7554/eLife.11284

**Published:** 2015-10-06

**Authors:** Pauline Formaglio, Rogerio Amino

**Affiliations:** Institute of Molecular and Clinical Immunology, Otto-von-Guericke University, Magdeburg, Germany; Unit of Malaria Infection and Immunity, Department of Parasites and Insect Vectors, Institut Pasteur, Paris, Franceroti@pasteur.fr

**Keywords:** malaria, sporozoite, gliding motility, skin, *Plasmodium*, mouse, other

## Abstract

In vivo imaging has revealed new details about how the malaria parasite enters the bloodstream.

**Related research article** Hopp CS, Chiou K, Ragheb DRT, Salman A, Khan SM, Liu AJ, Sinnis P. 2015. Longitudinal analysis of *Plasmodium* sporozoite motility in the dermis reveals component of blood vessel recognition. *eLife* **4**:e07789. doi: 10.7554/eLife.07789

**Image** Tracks (green) of malaria parasites moving through rodent skin

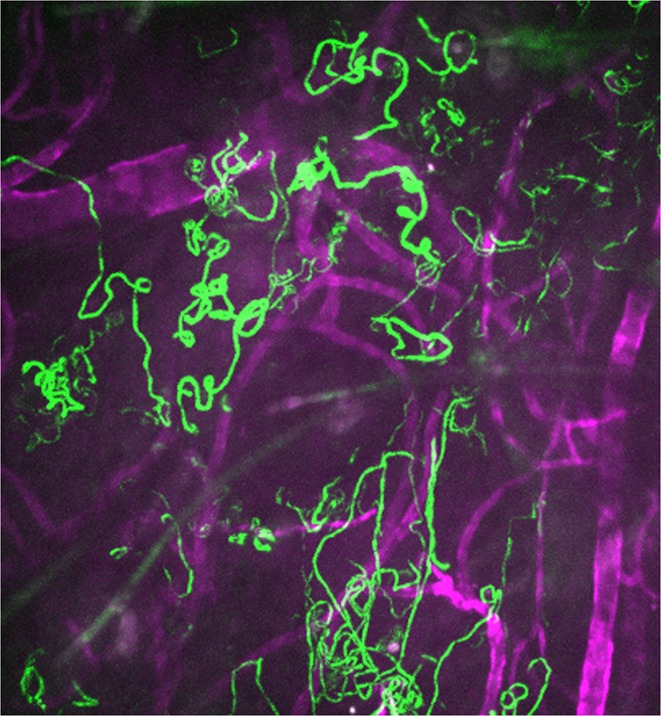

The parasites that cause malaria are spread by mosquitos, and many people think that these parasites go straight into the blood when a mosquito bites someone. However, studies have shown that these parasites are usually deposited in other parts of the skin, rather than in blood vessels. These parasites then can take between a few minutes and a few hours to reach the blood circulation (Sidjanski and Vanderberg, 1997; Amino et al., 2006; Yamauchi et al., 2007). Once in the blood the malaria parasites travel to the liver to multiply, and then start to infect red blood cells, which is when people develop symptoms of the disease.

In vivo imaging of malaria parasites in rodents that have been bitten by mosquitos has revealed how some parasites invade and complete their development inside skin cells, while others migrate across the skin to leave the bite site by actively entering into the lymph or blood vessels. The lymphatic route is a dead end for the parasites, but it is important for the activation of immune responses in the draining lymph nodes. The blood vessel route allows the parasites to enter the bloodstream and continue their development in the liver (reviewed in Ménard et al., 2013). Now, in *eLife*, Photini Sinnis of Johns Hopkins Bloomberg School of Public Health and co-workers – including Christine Hopp as first author – have used intravital microscopy to study in finer detail the movement of the malaria parasite in the skin of mice as they escape from the inoculation site (Hopp et al., 2015).

The first challenge for the malaria parasites is to find a blood vessel, which is not easy because blood vessels only occupy about 5% of the volume of the skin. Sinnis and co-workers – who are based at Johns Hopkins, the University of Pennsylvania and Leiden University Medical Center – show that the pattern of parasite migration does not resemble the Brownian-like random motion that is used by predators to locate abundant prey (Humphries et al., 2010). However, it remains to be determined whether the parasites rely on another type of random motion – such as the Lévy walk, which is better suited to finding sparse targets (Viswanathan et al., 1999) – or whether they are directed to blood vessels by chemical or physical cues.

When the parasites encounter a blood vessel, they often decrease speed as they circle around it (Amino et al., 2006) before they eventually enter the vessel en route to the liver (Vanderberg and Frevert, 2004). Using a syringe to inoculate parasites into the skin, which enables the observation of larger numbers of parasites, Hopp et al. confirm the existence of a slower and more constrained pattern of migration in the vicinity of blood vessels compared to the rest of the tissue (Figure 1 and Video 1). However, finding and interacting with a blood vessel is not sufficient to ensure that the parasite can enter the blood: this indicates the existence of another cue to help the parasite escape from the skin.

**Figure 1.**
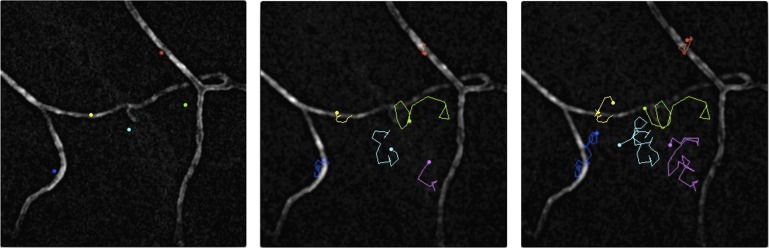
Malaria parasites moving near blood vessels in the skin. The paths followed by parasites in the dermis were tracked over a period of 145 seconds and then projected onto the image of blood vessels (white) from the same field of view. The paths followed by six different parasites are shown in six colours from their starting position (left) to their final position (right). The blue, yellow and red parasites display the slow and constrained ‘perivascular motility’ around blood vessels described by Hopp et al. On the other hand, the green, cyan and magenta parasites exhibit ‘avascular motility’, which is characterized by a faster migration and less confined trajectories. The imaged area measures 190 µm by 190 µm.

IMAGE CREDIT: PAULINE FORMAGLIO.

**Video 1.** Malaria parasites moving near blood vessels in the skin. See Figure 1 for details. Video credit: Pauline Formaglio.

Hopp et al. also dissect the behavior of two mutant parasites that are known to be less infective after they have been delivered into the skin (Coppi et al., 2011; Ejigiri et al., 2012). Both mutant parasites have defects in adhesion proteins. These mutants spend roughly the same amount of time in proximity to blood vessels as wild-type parasites do, but they are both much less able to invade blood vessels. Interestingly, the speed of the parasites seems to correlate (in a non-linear fashion) with their ability to invade blood vessels, but the meaning of this correlation is not clear.

Altogether, the work of Hopp et al. highlights that a variety of factors influences the ability of the parasites to invade blood vessels: some of these factors are related to the parasite and its motion, while others are related to the host and its blood vessels. A fuller understanding of these factors will help researchers working to develop new vaccine strategies to block parasites in the skin before they even reach the bloodstream.
